# Supplementary material for: A test of affect processing bias in response to affect regulation
Source: PLoS One. 2022 Mar 3;17(3):e0264758. doi: 10.1371/journal.pone.0264758 (PMC8893671; doi:10.1371/journal.pone.0264758)
Supplement: S1 Methods — (DOCX) [file pone.0264758.s001.docx]

**S1 Methods**

**Reliable Stimulus Subset Sampling**

In prior work we have shown that the difficulty of the affective decoding problem is largely a function of the distribution of training stimuli[1]. For example, training data that cluster at the polar extremes of an affective dimension confer a degree of simplicity to the decoding problem that does not necessarily reflect nature. We previously have described how image stimuli exhibit reliable properties of positive versus negative affect processing by measuring the degree to which the brain states induced by these stimuli cluster together. Stimuli exhibiting canonical two-class induction properties were labeled as part of the ‘Reliable Stimulus Subset’ (RSS) of the total stimulus set[1].

For clarity, we summarize the previously published RSS selection algorithm as follows. For each subject, i, for each stimulus, j, we evaluated the reliability of stimulus j based on the distribution of predictions made for this stimulus by the remaining set of study subjects (n=38). Each stimulus that exhibited prediction accuracy greater than chance according to the binomial distribution (n=38, *α*=0.05, h0: p[+]=0.5) was identified as ‘reliable’ and added to the subject’s reliable stimulus set, RSS_i_. We then conducted within-subject classification of the RSS using the i^th^ subject’s decoding model and reported accuracy on this dataset. Reporting RSS classification accuracy, in addition to classification accuracy on the full dataset, is important for understanding potential performance biases that may exist for curated datasets in which stimuli are hand-selected and, therefore, may possess artificially clustered normative affect scores, the existence of which we have previously reported[1,2].

**Minimal Image Preprocessing with fmriprep**

**Anatomical data preprocessing**

The T1-weighted (T1w) image was corrected for intensity non-uniformity (INU) with `N4BiasFieldCorrection` [@n4], distributed with ANTs 2.2.0 [@ants, RRID:SCR_004757], and used as T1w-reference throughout the workflow. The T1w-reference was then skull-stripped with a *Nipype* implementation of the `antsBrainExtraction.sh` workflow (from ANTs), using OASIS30ANTs as target template. Brain tissue segmentation of cerebrospinal fluid (CSF), white-matter (WM) and gray-matter (GM) was performed on the brain-extracted T1w using `fast` [FSL 5.0.9, RRID:SCR_002823, @fsl_fast]. Volume-based spatial normalization to one standard space (MNI152NLin2009cAsym) was performed through nonlinear registration with `antsRegistration` (ANTs 2.2.0), using brain-extracted versions of both T1w reference and the T1w template.

The following template was selected for spatial normalization: *ICBM 152 Nonlinear Asymmetrical template version 2009c* [@mni152nlin2009casym, RRID:SCR_008796; TemplateFlow ID: MNI152NLin2009cAsym],

**Functional data preprocessing**

For each of the 5 BOLD runs found per subject (across all tasks and sessions), the following preprocessing was performed. First, a reference volume and its skull-stripped version were generated using a custom methodology of *fMRIPrep*. Susceptibility distortion correction (SDC) was omitted. The BOLD reference was then co-registered to the T1w reference using `flirt` [FSL 5.0.9, @flirt] with the boundary-based registration [@bbr] cost-function. Co-registration was configured with nine degrees of freedom to account for distortions remaining in the BOLD reference. Head-motion parameters with respect to the BOLD reference (transformation matrices, and six corresponding rotation and translation parameters) are estimated before any spatiotemporal filtering using `mcflirt` [FSL 5.0.9, @mcflirt]. BOLD runs were slice-time corrected using `3dTshift` from AFNI 20160207 [@afni, RRID:SCR_005927]. The BOLD time-series (including slice-timing correction when applied) were resampled onto their original, native space by applying the transforms to correct for head-motion. These resampled BOLD time-series will be referred to as *preprocessed BOLD in original space*, or just *preprocessed BOLD*. The BOLD time-series were resampled into standard space, generating a *preprocessed BOLD run in MNI152NLin2009cAsym space*. First, a reference volume and its skull-stripped version were generated using a custom methodology of *fMRIPrep*. Several confounding time-series were calculated based on the *preprocessed BOLD*: framewise displacement (FD), DVARS and three region-wise global signals. FD and DVARS are calculated for each functional run, both using their implementations in *Nipype* [following the definitions by @power_fd_dvars]. The three global signals are extracted within the CSF, the WM, and the whole-brain masks. Additionally, a set of physiological regressors were extracted to allow for component-based noise correction [*CompCor*, @compcor]. Principal components are estimated after high-pass filtering the *preprocessed BOLD* time-series (using a discrete cosine filter with 128s cut-off) for the two *CompCor* variants: temporal (tCompCor) and anatomical (aCompCor). tCompCor components are then calculated from the top 5% variable voxels within a mask covering the subcortical regions. This subcortical mask is obtained by heavily eroding the brain mask, which ensures it does not include cortical GM regions. For aCompCor, components are calculated within the intersection of the aforementioned mask and the union of CSF and WM masks calculated in T1w space, after their projection to the native space of each functional run (using the inverse BOLD-to-T1w transformation). Components are also calculated separately within the WM and CSF masks. For each CompCor decomposition, the *k* components with the largest singular values are retained, such that the retained components' time series are sufficient to explain 50 percent of variance across the nuisance mask (CSF, WM, combined, or temporal). The remaining components are dropped from consideration. The head-motion estimates calculated in the correction step were also placed within the corresponding confounds file. The confound time series derived from head motion estimates and global signals were expanded with the inclusion of temporal derivatives and quadratic terms for each [@confounds_satterthwaite_2013]. Frames that exceeded a threshold of 0.5 mm FD or 1.5 standardised DVARS were annotated as motion outliers. All resamplings can be performed with *a single interpolation step* by composing all the pertinent transformations (i.e. head-motion transform matrices, susceptibility distortion correction when available, and co-registrations to anatomical and output spaces). Gridded (volumetric) resamplings were performed using `antsApplyTransforms` (ANTs), configured with Lanczos interpolation to minimize the smoothing effects of other kernels [@lanczos]. Non-gridded (surface) resamplings were performed using `mri_vol2surf` (FreeSurfer). Many internal operations of *fMRIPrep* use *Nilearn* 0.6.1 [@nilearn, RRID:SCR_001362], mostly within the functional processing workflow. For more details of the pipeline, see [the section corresponding to workflows in *fMRIPrep*'s documentation] (https://fmriprep.readthedocs.io/en/latest/workflows.html "FMRIPrep's documentation").

**Harmonized preprocessing across multiple studies for inter-study comparison of the neural encoding of affect processing**

We preprocessed all imaging data using a single harmonized pipeline across raw MRI data collected for three separate studies – the current study, which we refer to as the Causal Test of Emotion Regulation (CTER) study (n=40), as well as two other previously published studies – the Intrinsic Neuromodulation of Core Affect (INCA) study[3] (n=19) and another study which augmented the INCA study with additional subjects (n=31) that performed identical affect induction tasks to estimate the relative predictive fidelity of affective valence decoding models versus heart-rate deceleration, which we abbreviate as the HR study[2]. We reconstructed the decoding models for all subjects of all three studies using an identical image preprocessing and analysis pipeline. Preprocessing is described here.

All imaging data were acquired via a Philips 3T Achieva X-series MRI scanner (Philips Healthcare, Eindhoven, The Netherlands) with a 32-channel head coil. In brief, we acquired anatomic images with a MPRAGE sequence (matrix = 256 x 256, 220 sagittal slices, TR/TE/FA = 8.0844/3.7010/8°, final resolution =0.94 x 0.94 x 1 mm^3^. We acquired functional images with EPI sequence parameters: TR/TE/FA = 2000 ms/30 ms/90°, FOV = 240 x 240 mm, matrix = 80 x 80, 37 oblique slices, ascending sequential slice acquisition, slice thickness = 2.5 mm with 0.5 mm gap, final resolution 3.0 x 3.0 x 3.0 mm^3^. All MRI data were processed using AFNI (Version AFNI_19.1.04)[4]. Anatomical data were processed via skull stripping, spatial normalization to the MNI152 brain atlas, and segmentation via FSL[5] into white matter (WM), gray matter (GM), and cerebrospinal fluid (CSF). Functional images underwent the following processing sequence: despiking; slice-time correction; deobliquing; motion correction; transformation to the spatially normalized anatomic image; regression of the mean time course and temporal derivative of the WM and CSF masks as well as a 24-parameter motion model[6,7], spatial smoothing (6-mm FWHM Gaussian kernel); and scaling to percent signal change. Gray matter (GM) masks for each subject were created using anatomical segmentation. A group-level GM mask was constructed, separately for each study, incorporating only GM voxels present in ≥ 50% of the individual subject GM masks within that study.

**Comparing Neural Encodings of Affective Image Stimuli across Multiple Studies.**

We computed study-specific group-level encodings from fMRI data according to the methods described in the main manuscript (see Materials and Methods: Affect Processing State Encodings), restricting those encodings to the GM masks computed for each separate study. We then conducted comparisons, separately for the CTER study against the HR study and the CTER study against the INCA study. The rationale for these comparisons was that the HR and INCA studies validated the predictive fidelity of the encoded brain states against independent psychophysiological measures of, respectively, valence (measured via heart rate deceleration) and arousal (measured via skin conductance response). Comparison of inter-study similarity of the encoding hyperplanes was conducted in two ways: (1) all gray matter voxels and (2) all gray matter voxels in which the encoding values survived global permutation testing (p<0.05). We then modeled the shared variance between the two encodings using linear regression in which the CTER study’s encoding values were the independent variable and the comparison study’s encoding values (either HR or INCA) were the dependent variable.

**Supplemental Source Code and Data Availability**

The authors have made the source code used in this supplemental analysis publicly available: <https://github.com/kabush/CMPhaufe>. The authors have also made the derivative activation maps necessary to execute this code available via the project’s Open Science Framework Repository: <https://osf.io/yn4vq/>.

**Supplemental References**

1. Bush KA, Gardner J, Privratsky A, Chung M-H, James GA, Kilts CD. Brain States That Encode Perceived Emotion Are Reproducible but Their Classification Accuracy Is Stimulus-Dependent. Frontiers in Human Neuroscience [Internet]. 2018 Jul 2 [cited 2018 Jul 25];12. Available from: https://www.frontiersin.org/article/10.3389/fnhum.2018.00262/full

2. Wilson KA, James GA, Kilts CD, Bush KA. Combining Physiological and Neuroimaging Measures to Predict Affect Processing Induced by Affectively Valent Image Stimuli. Sci Rep. 2020 Dec;10(1):9298.

3. Bush KA, Privratsky A, Gardner J, Zielinski MJ, Kilts CD. Common Functional Brain States Encode both Perceived Emotion and the Psychophysiological Response to Affective Stimuli. Scientific Reports [Internet]. 2018 Dec [cited 2018 Oct 18];8(1). Available from: http://www.nature.com/articles/s41598-018-33621-6

4. Cox RW. AFNI: software for analysis and visualization of functional magnetic resonance neuroimages. Computers and Biomedical research. 1996;29(3):162–73.

5. Jenkinson M, Beckmann CF, Behrens TEJ, Woolrich MW, Smith SM. FSL. NeuroImage. 2012 Aug;62(2):782–90.

6. Power JD, Barnes KA, Snyder AZ, Schlaggar BL, Petersen SE. Spurious but systematic correlations in functional connectivity MRI networks arise from subject motion. NeuroImage. 2012 Feb;59(3):2142–54.

7. Power JD, Schlaggar BL, Petersen SE. Recent progress and outstanding issues in motion correction in resting state fMRI. NeuroImage. 2015 Jan;105:536–51.
