# Supplementary figures and images for: A test of affect processing bias in response to affect regulation
Source: PLoS One. 2022 Mar 3;17(3):e0264758. doi: 10.1371/journal.pone.0264758 (PMC8893671; doi:10.1371/journal.pone.0264758)

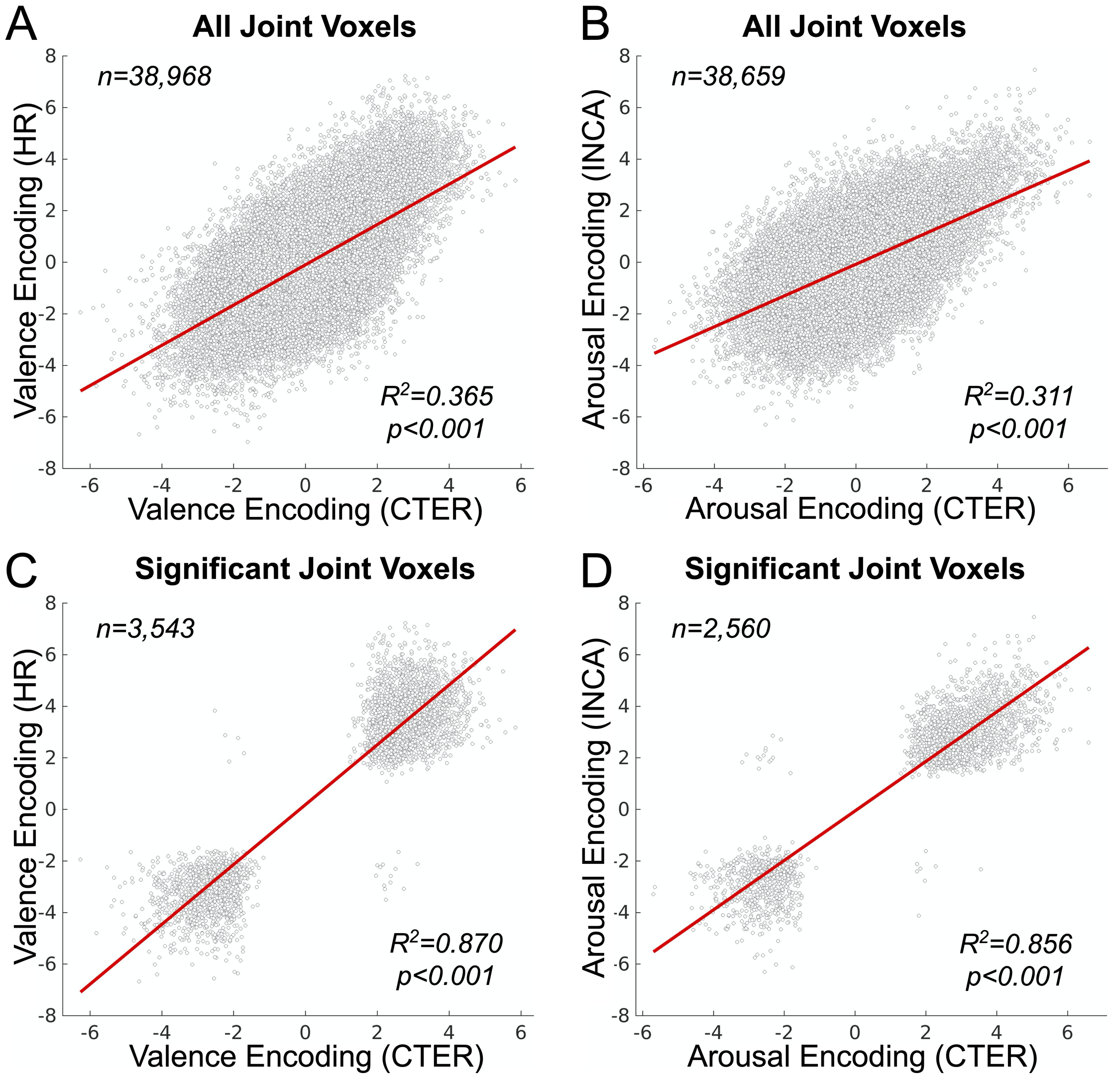

Supplement: S1 Fig — (Top Row): Inter-study comparison that includes the encoding values of all joint GM voxels shown for (A) affective valence processing and (B) affective arousal processing. (Bottom Row): Inter-study comparison that include encoding values for only those joint GM voxels that survive global permutation significance testing (p < .05) for (C) affective valence processing and (D) affective arousal processing. Voxel-wise relationships are depicted as gray circles. The regression fit of the voxel-wise relationships is represented by the bold red line in each subplot. Total surviving joint voxels for each comparison are provided in the top left of each subplot. Inter-study shared variance is provided in the bottom right of each subplot. P-values refer to the significance of the regression fit’s linear coefficient (t-test, α = 0.05). (TIF) [file pone.0264758.s002.tif]

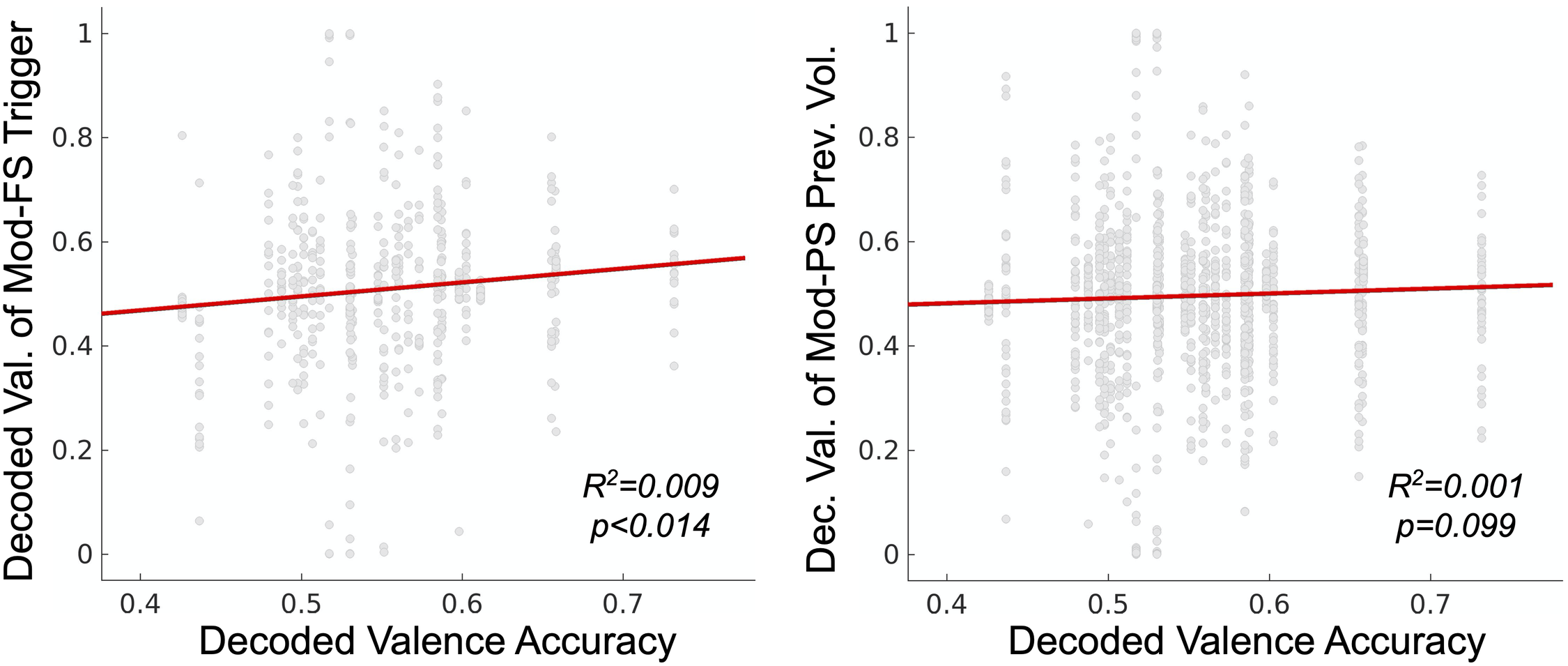

Supplement: S2 Fig — (Left) The magnitude of real-time self-induced positive affect processing (Mod-FS trials) according to the method of iteratively reweighted least squares versus. The measure of interest is Platt-scaled decoded valence observed at the moment of stimulus triggering. The fixed effect is the valence decoding model accuracy (measured according to the Full stimulus Set). Post-hoc decoding accuracy, which potentially reflects real-time decoding and, therefore, self-induction performance, was found to have a small (R2 = 0.009) but significant positive effect on the decoded valence at the moment of real-time stimulus triggering (β = .23; p = 0.014; t-test; α = .05; h0: β = 0). (Right) The effect of post-hoc decoding model accuracy on the magnitude of random affect processing occurring in the fMRI volume acquired immediately prior to passive image stimulation (Mod-PS trials). No significant effects were observed (β = .09; p = 0.099; t-test; α = .05; h0: β = 0). (TIF) [file pone.0264758.s003.tif]
