# Supplementary material for: A test of affect processing bias in response to affect regulation
Source: PLoS One. 2022 Mar 3;17(3):e0264758. doi: 10.1371/journal.pone.0264758 (PMC8893671; doi:10.1371/journal.pone.0264758)
Supplement: S1 Table — IAPS identification numbers listed separately for Mod-PS and Mod-FS trial types. (DOCX) [file pone.0264758.s004.docx]

**S1 Table. International Affective Picture Set Image Identification Numbers**

| **Trial Type** | **Identification Numbers** |
| --- | --- |
| **Mod-PS** | 8510, 9421, 3350, 7502, 9908, 3266, 3061, 6821, 9910, 3140, 2799, 2717, 8420, 7230, 3168, 2800, 8503, 4520, 9253, 5460, 9250, 5830, 4608, 8380, 9901, 2208, 2160, 9400, 7260, 5825, 8300, 4660, 4640, 8210, 9500, 9040, 7405, 9412, 2075, 3131 |
| **Mod-FS** | 8034, 9419, 2071, 8490, 9570, 9300, 5480, 3301, 9830, 8170, 4680, 3215, 9183, 8370, 3225, 9921, 3064, 4599, 7350, 5450 |
