# Supplementary material for: A test of affect processing bias in response to affect regulation
Source: PLoS One. 2022 Mar 3;17(3):e0264758. doi: 10.1371/journal.pone.0264758 (PMC8893671; doi:10.1371/journal.pone.0264758)
Supplement: Attachment — Submitted filename: Response_to_Editorial_Review.docx [file pone.0264758.s007.docx]

**Response to Prior Editorial Review**

**This manuscript was previously submitted to PLOS Biology (manuscript number PBIOLOGY-S-21-00981) and was declined based on review by an Academic Editor. We wish to respond to prompt reconsideration of its suitability for publication; we reiterate each of this editor’s comments below and describe our specific responses (bold font).**

-It's not clear that what the decoder is tracking is really affective state. It could be tracking engagement of a particular strategy or type of appraisal, for example. It could also be associated with expectations: If participants are aware of any experience (even if not "positive") that correlates with the "positive state", they may expect to feel more positively. Thus, the conditional presentation of stimuli on the neural measure is not really a clean causal manipulation of affective state. The design seems susceptible to demand characteristics. Demonstrating clear effects of the "affective brain state" on valence-related EMG measures could help, but this does not appear to be a focus here.

**After thoughtful consideration we now offer three responses to this overall comment.**

1) **The decoding models were trained to maximize generalized prediction performance (i.e., minimize error in the prediction of novel stimuli). Model performance was evaluated against this specific criterion and found to be significant at both the group level and generally significant at the individual level. This result strongly implies that the conveyed affect was experienced by the subjects though we acknowledge the possible co-occurrence of other cognitive processes as further responses to the image stimuli. Indeed, image stimulus-induced affect processing (as was used to train the models) confounds the affective valence and arousal processing subsystems for any stimulus having non-neutral affective properties simultaneously for both dimensions. Despite this potential confound, each underlying dimension of affect processing was captured by the decoding models. Moreover, the perceived affective experiences of these processes were independently validated based on their psychophysiological response corollaries. It is exactly our use of decoding models, trained to maximize generalization performance, that mitigates this confound. We have clarified our reporting of these carefully controlled validations (see both Sections Psychophysiological Response Validation of Affect Processing Induction via Image Stimuli and Validation of Affect Decoding using Novel Stimuli) to specifically support the effectiveness of our decoder approach.**
2) **We have also clarified our reporting of the explicit emotion regulation validation task results (based on known regulation strategies and goals), see Figure 6, in order to confirm (via both valence decoding and independent facial EMG) the validity of affective decodings captured during explicit emotion regulation, which further illustrates that the decoding approach mitigates demand characteristic confounds.**
3) **As a further response we have revised the Discussion section to fully acknowledge both the limitations of decoding models as well as the limitations of this real-time MRI approach, particularly our inability to capture self-reported responses at the moment of triggering, which would isolate and enable the ability to quantify the role of demand characteristics in triggering the Mod-FS trials (see Figure 2).**

-There is no characterization of what the affective brain state is or which kinds of activity are driving it, and no attempt in the manuscript at construct validation.

1) **In response, we have revised the manuscript to include a full characterization of the affective brain state, see Figure 4. We have also added supplemental sections that quantify the relationship between the brain states derived in this work with those brain states identified in earlier reports that investigated both the convergent validity of these brain states with respect to psychophysiological measures[1,2] as well as the construct validity of the brain states in relation to the circumplex model[3].**

-The style of the manuscript detracts from the main messages at some points, with a great deal of focus on some details of mixed-effects models that may not be essential to the main story. The figures are limited in informativeness.

1) **We are inclined to report all models according to the following sequence of validation steps presented in the Results section of the manuscript and prior to our reporting of the main finding: 1) the affect processing task successfully induced a congruent perceived affective experience; 2) the decoding models generalize to brain states induced by stimuli not previously used to fit the models; 3) the brain states being decoded have convergent validity with independent psychophysiological measures of affect processing and also exhibit construct validity with the circumplex model of affect; 4) the real-time system triggered image stimulus presentation when and while the subject experienced positively valent states (as measured via neural decoding); and, 5) induced positive valence brain states positively biased the valence processing of subsequent image stimuli.**
2) **Without specific information as to which Figures are perceived to have limited information, we are inclined to provide figures that illustrate aspects of our approach or results to best convey the findings of the study. Our judgement on this matter is informed by our prior experience in addressing confusion or concerns related to the construction and validation of decoding models (e.g., the exact stimuli used, their affective properties, and the sequencing of training versus application of the decoding models in the experiment).**

**References**

1.	Bush KA, Gardner J, Privratsky A, Chung M-H, James GA, Kilts CD. Brain States That Encode Perceived Emotion Are Reproducible but Their Classification Accuracy Is Stimulus-Dependent. Frontiers in Human Neuroscience [Internet]. 2018 Jul 2 [cited 2018 Jul 25];12. Available from: https://www.frontiersin.org/article/10.3389/fnhum.2018.00262/full

2.	Wilson KA, James GA, Kilts CD, Bush KA. Combining Physiological and Neuroimaging Measures to Predict Affect Processing Induced by Affectively Valent Image Stimuli. Sci Rep. 2020 Dec;10(1):9298.

3.	Bush KA, Privratsky A, Gardner J, Zielinski MJ, Kilts CD. Common Functional Brain States Encode both Perceived Emotion and the Psychophysiological Response to Affective Stimuli. Scientific Reports [Internet]. 2018 Dec [cited 2018 Oct 18];8(1). Available from: http://www.nature.com/articles/s41598-018-33621-6
